# Time series analysis as input for clinical predictive modeling: Modeling cardiac arrest in a pediatric ICU

**DOI:** 10.1186/1742-4682-8-40

**Published:** 2011-10-24

**Authors:** Curtis E Kennedy, James P Turley

**Affiliations:** 1 Department of Pediatrics, Baylor College of Medicine, 6621 Fannin, WT 6-006, Houston, TX 77030, USA; 2 The University of Texas School of Biomedical Informatics, 7000 Fannin, Suite 600, Houston, TX 77030, USA

## Abstract

**Background:**

Thousands of children experience cardiac arrest events every year in pediatric intensive care units. Most of these children die. Cardiac arrest prediction tools are used as part of medical emergency team evaluations to identify patients in standard hospital beds that are at high risk for cardiac arrest. There are no models to predict cardiac arrest in pediatric intensive care units though, where the risk of an arrest is 10 times higher than for standard hospital beds. Current tools are based on a multivariable approach that does not characterize deterioration, which often precedes cardiac arrests. Characterizing deterioration requires a time series approach. The purpose of this study is to propose a method that will allow for time series data to be used in clinical prediction models. Successful implementation of these methods has the potential to bring arrest prediction to the pediatric intensive care environment, possibly allowing for interventions that can save lives and prevent disabilities.

**Methods:**

We reviewed prediction models from nonclinical domains that employ time series data, and identified the steps that are necessary for building predictive models using time series clinical data. We illustrate the method by applying it to the specific case of building a predictive model for cardiac arrest in a pediatric intensive care unit.

**Results:**

Time course analysis studies from genomic analysis provided a modeling template that was compatible with the steps required to develop a model from clinical time series data. The steps include: 1) selecting candidate variables; 2) specifying measurement parameters; 3) defining data format; 4) defining time window duration and resolution; 5) calculating latent variables for candidate variables not directly measured; 6) calculating time series features as latent variables; 7) creating data subsets to measure model performance effects attributable to various classes of candidate variables; 8) reducing the number of candidate features; 9) training models for various data subsets; and 10) measuring model performance characteristics in unseen data to estimate their external validity.

**Conclusions:**

We have proposed a ten step process that results in data sets that contain time series features and are suitable for predictive modeling by a number of methods. We illustrated the process through an example of cardiac arrest prediction in a pediatric intensive care setting.

## Background

Roughly 1-6% of children being cared for in an ICU will experience a cardiac arrest while in the ICU [1,2]. Many of these arrests occur because their vital signs deteriorate to the point where they enter a state of progressive shock [3-5]. These arrests happen despite the fact that they are being continuously monitored by ECG, pulse oximetery, and frequent blood pressure measurements. While there are tools that help identify patients in a non-intensive care setting that are at risk of arrest or have deteriorated to the point where they need to transfer to an ICU[6-21], there are no equivalent tools to identify which patients are likely to arrest in an intensive care setting. That is not to say that ICU environments are devoid of useful tools that provide decision support: systems such as VISICU and BioSign/Visensia [22-26] provide an added level of safety to the ICU environment by enabling remote monitoring and providing automated rule checking and high-specificity alerting for deteriorations that occur across multiple channels. While these tools are excellent for detecting deteriorations, they still are largely lacking in their ability to predict specific adverse outcomes. The goal of this study is to develop a framework for building prediction models that use time series data and can serve as the foundation for tools that can evaluate for specific consequences of a deterioration, with the ultimate goal of augmenting existing systems with the ability answer questions like "Who is most likely to arrest?" in an ICU environment.

There are over 13,000 tools available to help clinicians interpret the data they are presented [27]. Almost all of these tools have been designed so that they can be manually used. A tool's adoption typically depends on a balance between how easy it is to use and what the information content of the tool is [28,29], so tools that are built to be manually used are constrained to a relatively small number of variables in order to achieve adequate simplicity. As a result, input variables have typically been restricted to a multivariable data paradigm where each variable is represented by a single value. A consequence of this strategy is that useful trend information cannot be incorporated into a model unless it is explicitly encoded as a variable. Of course doing this would add complexity to the task, so it is therefore rarely done.

As healthcare is transitioning from manual processes to electronic ones, it is becoming increasingly easy to automate the processes of data collection and analysis. In an automated system, there is no longer a need to remain constrained to a multivariable data paradigm in order to achieve simplicity at the user level. Clinical studies using time series analysis has been undertaken in a number of settings [30-41], but thus far has been relatively limited in scope, tending to focus on interpretation of a single analytic method rather than incorporating multiple analytic methods into a more robust modeling paradigm.

The purpose of this article is to describe a method for developing clinical prediction models based on time-series data elements. The model development process that we are presenting is novel to clinical medicine, but the individual steps comprising the process are not. Our intention is to provide not only the description of the method, but the theoretical basis of the steps involved. We are demonstrating the application of this process in an example of cardiac arrest prediction in a pediatric intensive care unit. It is our hope that we describe the steps of the process and their theoretical basis clearly enough that the methods can be extended to other domains where predictions based on time-series data is needed.

## Introduction

In order to ensure that the concepts in this article can be understood by clinician and nonclinician alike, we will provide four brief overviews of the core concepts that form the foundation of this article. First, we will describe how the growth of data has impacted medicine and some of the strategies that have evolved to manage this growth. Second, we will review a few relevant concepts that relate to statistical analysis and modeling, with special focus on multivariable versus time series data paradigms. Third, we will specifically discuss clinical prediction models: their utilities, their limitations, and considerations for improvements. Finally, we will review the rationale behind selecting cardiac arrest in a pediatric ICU (PICU) as the example to illustrate the process, and we will provide a brief overview of the physiologic principles that serve as the theoretical basis for our prediction model.

### Data in Medicine

Medical care has existed since long before diseases were understood at a scientific level. Early medical care was characterized more by art and religion than by science as we know it today [42]. The transition of medicine from an art to a science is based on the accumulation of data, and the information, knowledge, and wisdom that has been derived from it. While this transition has improved outcomes, there is a side effect of the data: information overload [43-46]. Currently, the amount of data in the medical field is so extensive and is growing so fast that is impossible for any single person to utilize it all effectively. In order to utilize data, it must be interpreted in context (transforming it into information) and evaluated by the user [47]. This process requires substantial cognitive resources and is time consuming. In an attempt to address this problem, at least two strategies have been employed: specialization and computerized support [43,48]. Specialization allows clinicians to focus their efforts on a narrow field where they become expert in a relatively small group of related diseases. In doing so, they reduce their educational burden to a point where they can "afford" the cost of training and staying current in their specialty. In fact, some specialties have even reached the point where subspecialization is required in order to stay abreast of the latest trends [49]. There is a fundamental limitation to specialization as a means to cope with excessive amounts of data or information: a more robust solution to the problem is needed. Ideal properties of the new solution should include: scalability [50] (it can continue to grow indefinitely), flexibility [50,51] (it can be used for a number of purposes), explicit and accurate [51] (it relies on objective parameters), and automaticity [52] (it functions independent of frequent supervision). Computer technology possesses these characteristics, and the field of informatics has been born out of effort to utilize computer based solutions to automate the transformation of data to information in the healthcare setting [53,54]. These solutions come in many forms, ranging from aggregating knowledge available on a given disease to informing clinicians when tests or treatments violate parameters deemed to be unsafe [55,56]. One of the fundamental goals of this article is to describe a method that can be automated as a computer based solution to help inform clinicians of a patient's risk of cardiac arrest using trend information that would otherwise require manual interpretation. Since clinicians cannot continuously check the risk of cardiac arrest for all patients they are caring for, we are attempting to leverage information from data that would otherwise be left unanalyzed in the current "intermittent check" paradigm.

### Statistical Analysis and Modeling

Of course, medicine is not the only field where data has become so abundant that it is impossible to understand it all. Compared to fields such as physics and astronomy, medicine is in a relative state of adolescence. When presented with an abundance of data, the first priority is to understand what the data represent. This process of gaining an understanding is based on statistical analysis [57-59]. Depending on the information needs, data can be analyzed in a number of ways to provide a range of understandings. For instance, a univariable analysis [60] of "heart rate" provides an understanding of what the most common heart rate is, the range of heart rates, and how the range is distributed. A multivariable analysis [61] that includes "heart rate" as a variable can provide an understanding of how heart rate relates to temperature or blood pressure. A time series analysis [62] of "heart rate" can provide an understanding of how the heart rate changes different times of the day. The statistical methods for analyzing the data differ fundamentally for time series data since a single variable is represented by multiple values that vary depending on the time they represent. Univariable and multivariable statistics, on the other hand, rely on a single value per variable for each case. Also, time series data elements are assumed to correlate to adjacent data elements [62], whereas this type of correlation can interfere with univariable and multivariable analysis [63,64].

Whereas univariable and multivariable data analysis informs the user of the distribution of a variable across a population and how the variable relates to other variables, time series analysis informs the user of how a variable relates to itself. In particular, time series analysis provides two types of information about a variable of interest: trends and seasonality [65]. The distinction between the two is that univariable and multivariable analyses aim to describe the static properties of a variable, whereas the aim of a time series analysis is to describe its dynamic properties over time. Knowing an airplane is 10 feet off the ground with the nose angled up and is at full throttle are static variables that would suggest a plane is taking off. However, knowing that over the last five seconds the elevation was 150 feet off the ground, then 140, then 120, then 90, and then finally 60 feet off the ground changes the interpretation of the multivariable data to suggest that the plane is about to crash. The addition of the trend features for the altitude changes the interpretation of the static data about height, pitch and thrust significantly.

Statistical analysis provides a systematic and standardized process of characterizing data so that it can be understood in the context that it is being analyzed. Modeling endeavors also require a systematic approach, but the range of options is more varied than in statistical analysis [66,67] since the products of analyses are often used as "building blocks" for a model. It is not uncommon for models to draw on elements from more than one type of analysis in making a prediction. One example of this hybrid technique is the *time-course approach *to microarray analysis [68,69]. As an example of this approach, the expression levels of twenty different genes are measured to determine their activity in two classes of cancer. If it were to stop here, this would be a basic multivariable model. However, the expression levels of these same twenty genes are measured repeatedly under different conditions and at different points in time. Under the standard multivariable model that used baseline expression levels of the twenty genes, it is impossible to tell which genes determine cancer class. However, by adding the behavior over time in the different nutrient environments, the different classes of cancer can be distinguished from one another. This is a well established technique for genomic modeling. The technique is based on a paradigm that utilizes time series data elements in a multivariable data format. In multivariable statistical analysis, a high degree of correlation between independent variables (known as multicollinearity - an inherent feature of time series data) can invalidate the results of the analysis by invalidating the calculations relating to the analysis of the independent variables as unique components [63,64]. However, when modeling is focused on the relationship between the dependent variable and the aggregate of all independent variables (without trying to measure the effects of the independent variables themselves), this multicollinearity is permissible [70].

### Clinical Prediction Models

For centuries, models have been used to demonstrate our knowledge about the world in which we live. They help us share our understandings about the observations we make, and they help us anticipate what is to come. In medicine, scoring tools are a class of models that combine multiple data elements, weight them according to their correlation with the outcome of interest, and output a score that can be used in a number of ways. Individual scores can be used to make predictions that can help guide treatment decisions and communications with patients and families. As an example, medical emergency teams use scoring tools to identify high risk patients that merit transfer to a higher acuity unit [6-9,13-21]. Grouping scores allows standardized comparisons between two or more entities by providing a risk-based adjustment to the outcome of interest [10-12,71,72].

Almost all clinical models are built on multivariable regression or a regression-like approach that evaluates a number of candidate input features (variables) and measures their individual correlation with the outcome of interest. The strength of the correlation is used to assign points for each of the included variables, with more points being assigned for highly correlated variables and for greater deviation from the variable's normal value. Finally, points attributable to each feature are summed together to provide the composite score that provides an estimate of the net effect of all the features combined. To illustrate, the Pediatric Risk of Mortality (PRISM) score [11,12] assigns a child who has a heart rate of > 150 beats per minute (bpm) 4 points for the abnormal heart rate. Heart rate is not the strongest predictor of death though - plenty of children admitted to the PICU have heart rates > 150 bpm during the first 24 hours and survive. However, if the child's pupils are fixed and dilated (evidence of severe brain dysfunction), they get 10 points for pupillary reaction: kids that have this degree of brain dysfunction are much more likely to die than those that have a high heart rate - thus the higher score. After points are assigned for each of the variables, all of the points are added together to generate the overall PRISM score. The combined score is then entered into an equation that provides the user with the probability of death during the PICU stay.

Since most of these scoring tools have been built using a multivariable data paradigm that is constrained to a single value per variable, they are generally limited to evaluating a static state at one point in time. They are unable to characterize an extremely important type of information: *trends*. In order to evaluate a dynamic state over multiple points in time, a time series data paradigm is required. However, since most scoring tools weight their independent variables differently based on regression coefficients, they are prohibited from using data with high degrees of multicollinearity and are therefore unable to use time series data.

While multivariable models prevail in the setting of clinical prediction tools, there are small but growing number of medical models based on time series data. These models have been used in a number of settings [73,74] ranging from imputation strategies for missing data [75] to analysis of beat-to-beat variability in heart rate as a way to discriminate survivors from nonsurvivors [40,41]. However, unlike the multivariable based scoring tools that tend to employ a spectrum of independent variables, most medical models that use time series data have restricted their focus to the time series features of a limited number of independent variables.

Finally, there is the concept of using the results of multiple models as latent independent variables in their own right. While there is precedent for this is in financial and weather forecasting disciplines [76,77], it is not a common practice in medicine. There are plenty of examples of studies that compare performance of one model to another, but studies that combine two or more predictive models to arrive at a new prediction are sparse. A general observation noted in our review of these types of studies is that if two or more models are based on similar data, then one of the component models often dominates and there is little effect of adding the second model. However, if the models are based on disparate data, the resultant model typically performs better than either of the component models in isolation.

### Inpatient Cardiac Arrest as the Example to Illustrate the Process

In order to build a clinical prediction model that combines the traditional multivariable data elements with the time series data elements, we sought out a problem space that had the following characteristics: 1) target problem has a known relationship to variables measured in a time series fashion; 2) measured variables are abundantly available; 3) time series elements are likely to help predict the target problem. We selected "cardiac arrest in a pediatric intensive care unit" as our target for a number of reasons. First, we were able to identify all cases of cardiac arrest easily since they are recorded on specialized code sheets. Second, standardized criteria [78] can be used to isolate true cardiac arrests from other events that get documented on the code sheets. Finally, cardiac arrest is a significant, life threatening condition that predictably results when a patient's vital signs deteriorate beyond a point of compensation. As vital signs deteriorate, patients progress from a state of normalcy, to a state of compensated shock, to a state of uncompensated shock, and finally to cardiac arrest. Progressive shock is one of the leading causes of pediatric cardiac arrest [3]. Given that shock can be characterized by vital signs (establishing their plausible association to cardiac arrest) and vital signs are automated and ubiquitously available in pediatric intensive care settings, we felt this was an appropriate example on which to illustrate the process. Furthermore, since shock can often be reversed with treatment, we believe there is a possibility of real world application of the example.

After establishing that cardiac arrest fits the desired criteria, the spectrum of possible conditions that can lead to cardiac arrest must be considered. In reviewing the literature for inpatient cardiac arrest, we determined that patients arrest due to a number of other causes [3], including intrinsic arrythmias that can send a patient into immediate cardiac arrest, and unexpected events that can result in cardiac arrest in a matter of minutes, such as sudden uncontrollable bleeding, unplanned removal of life support devices such as ventilators or endotracheal tubes [79], and embolic phenomena such as pulmonary embolism. The list of possible causes is extensive, but almost all causes not attributable to progressive shock share a common feature: they lead to arrest very rapidly. Also from our review of inpatient cardiac arrest literature, we discovered that shock is usually insidious in onset and is characterized by deterioration over minutes to hours, whereas the other causes of arrest are characterized by deterioration over seconds to minutes. Finally, shock can be characterized by vital sign data, while other causes of arrest are not so easy to characterize. Given the slower nature of the progressive shock process affords a greater amount of data than the other processes, we felt it appropriate to constrain the example model to parameters that relate to shock.

The fundamental feature of shock is that the body's need for energy is not being supplied in sufficient quantities. By far, the most frequent cause of shock in the pediatric intensive care setting is one of insufficient oxygen delivery to the tissues [80]. Shock can be described from a perspective of supply and demand. On the supply side, oxygen delivery is a process that is dependent on: hemoglobin, oxygen, and blood flow [80]. Hemoglobin and oxygen can be measured directly. Measuring a patient's blood flow, on the other hand, is not commonplace. However, blood flow is a function of heart rate and the stroke volume associated with each heartbeat. Heart rate is measured directly, but again, stroke volume measurements are uncommon. For a fixed vascular resistance, though, stroke volume is proportional to the pulse pressure (the difference between systolic and diastolic blood pressure readings) [81]. The pulse pressure can be directly measured. One other nuance regarding oxygen delivery is that it is dependent on the pressure gradient across the tissue bed, so the gradient between the mean arterial pressure and the central venous pressure is important. Mean arterial pressure can be determined from systolic and diastolic blood pressures. Central venous pressure, on the other hand, is only obtained in a relatively small fraction of the population. Not having this value readily present for the majority of the population is a potential obstacle to being able to model cardiac arrest due to progressive shock.

When examining the variables that relate to the supply of oxygen to the body, most adhere to the desired features of being automatically collected by the monitors, reliably measured, and ubiquitous in the pediatric intensive care population. Oxygen demand depends primarily on temperature and level of activity. Temperature is measured directly, but the method of measurement determines the accuracy of the reading: core temperatures esophageal or rectal probes tend to be more accurate than oral or axillary readings [82]. Furthermore, some measurement modalities are integrated into the physiologic monitoring system, which has two implications: 1) it allows for automated capture, which can also achieved with an electronic medical record; and 2) it allows for continuous measurement, whereas others typically do not. Therefore, care should be exercised when using temperature as a variable to characterize oxygen demand. This introduces another potential obstacle for successfully modeling cardiac arrest. Level of activity is comprised of a host of factors ranging that can include factors such as the work of breathing, digestion, presence of chills and rigors, seizure activity, and a number of other conditions. It is generally not measured in an objective, quantitative fashion, and again, not being able to incorporate it into the modeling process poses a risk to diminishing model performance.

Although we identified at least three potential weaknesses, we have established that there are still a number of variables that are time series in nature and directly relate to the physiology of shock. Despite the risk of not being able to generate an *ideal *model, we nonetheless felt there was a sufficient amount of data to determine whether the addition of time series data elements influence model performance, as compared to baseline multivariable analysis.

## Methods [& Results]

In order use time series data in a clinical predictive modeling paradigm that is based on a multivariable data format we needed to accomplish three fundamental tasks: 1) characterize models that utilize time series data to perform classification; 2) explicitly represent the candidate features that determine the target of interest in both multivariable and time series fashions, including: a) specific measurement modalities; b) windows of observation; c) resolution of observations; and d) computations required to derive the time series features such as slopes and intercepts; and 3) create the modeling data sets using the candidate features in a data structure supported by the modeling algorithm.

The method we are proposing is listed below as a series of steps. In order to maintain continuity of focus between the method and the results, we will begin each section by identifying the task and providing a general description of the concepts and theories that we are applying. As the result for each step, we illustrate the step using the specific case of modeling cardiac arrest in a pediatric intensive care unit. [The illustration is indented and placed in brackets.]

### Determine Model Characteristics

In order to discover which what characteristics and properties could potentially suit our needs, we examined models from a variety of disciplines that use time series data. Starting at the broadest level, we initially searched web-accessible articles for "time series" and "prediction model." A basic exploration of the qualitative properties of the resultant hits produced several observations that provided focus for subsequent analyses.

[The first observation was that some models rely on raw statistical associations while other models utilize explicit equations for mathematical or physical properties. For instance, financial models tended to have a more statistical focus while engineering models tended to provide mathematical representations for the phenomena being studied. The second observation was that the majority of models utilize information from past events to predict future events. While measures of seasonality are germane to many areas of medical predictive modeling, they do not apply to cases where initial or singular events are the target, which is the case in this study. The final observation was that "pattern recognition" and "classification" tasks more precisely describe the focus of our study.]

Refining our screening query to "time series" and either "pattern recognition" or "classification" we obtained a more homogenous group of studies, including a greater fraction from medically related fields.

[However, clinical models were still lacking, and strategies to predict initial events were rare. One class of studies that seemed to hold promise was based on *time course *analysis [68,69]. Frequently used in genomic classification tasks, this strategy has been used to classify diseases where less than 100 samples were available for training but thousands of candidate variables were being analyzed. The method relies on defined variables (gene expression levels) under defined conditions (exposure to different agents) at defined times of measurement (baseline and several post-exposure times). The methods share many of the properties we desire for modeling cardiac arrest, and we therefore focused our efforts on adapting this strategy as a template for our work.]

### Select Candidate Features

Selecting the set of variables to serve as candidate features that will discriminate between different classes is of key importance in modeling. A combination of approaches, including literature review, concept mapping [83,84], and statistical analysis are several methods that can be used to identify plausible features. Ideally, features should describe the target, correlate with the target, or have other plausible associations with the target.

**Figure 1:**
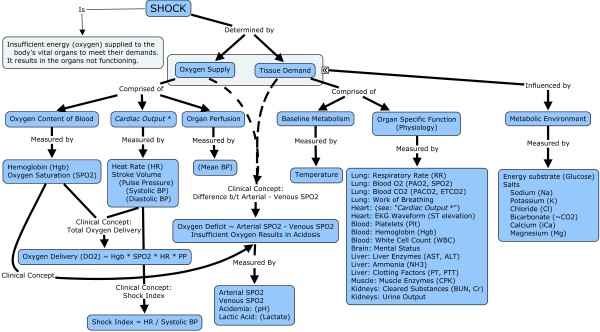
**Overview of shock**. Determinants of shock include variables that represent supply, demand, measures of end organ function, and modulators of the metabolic environment. Clinically useful concepts that are not explicitly measured but directly relate to supply and demand of oxygen are represented as explicitly calculated latent variables.

**Figure 2:**
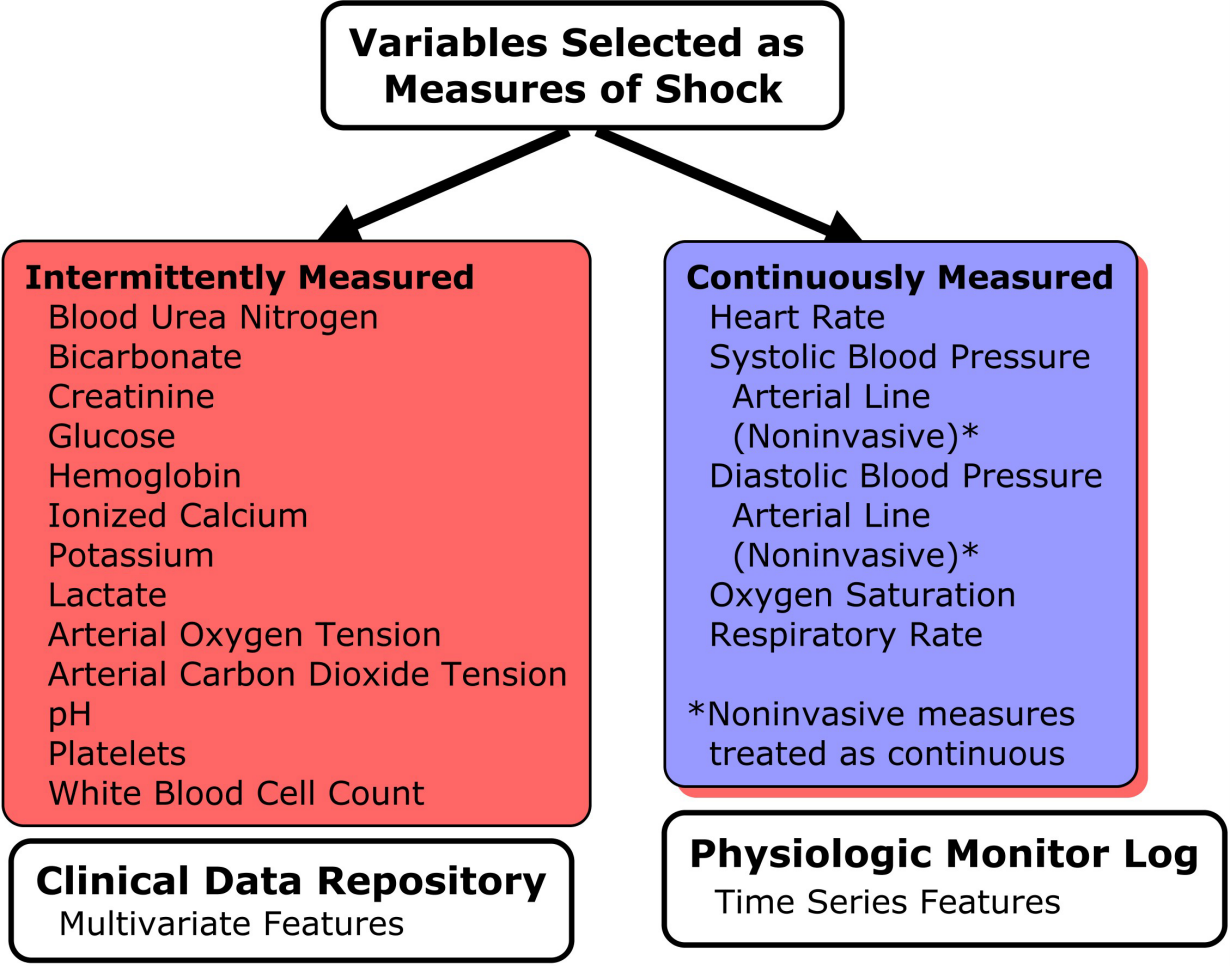
**Determinants of shock: variable source and data type**. Physiologic variables selected as determinants of shock. Each variable has been matched to a source that contains the raw measurement. For measurements with more than one source, the preferred source was used for the match.

[Since shock states are characterized by imbalances between supply and demand, and since neither is constant, there is no established variable or combination of variables that can be used to identify the threshold at which to define shock. However, we can define a set of variables that semiquantitatively represent supply and demand, and we can measure several markers of anaerobic metabolism, which takes place during shock states where demand exceeds supply. In addition to the direct determinants of shock, there are associated variables that may modulate the baseline risk of cardiac arrest: the overall metabolic profile, comprised of various salts in the body is one modulator, and the functional status of the organs of the body is another. These modulators are often measured as laboratory values. In order to select candidate features for modeling cardiac arrest, we generated a list of variables based on concept mapping [83,84] and literature review that are relevant to shock and matched them with data that was electronically available. The concept map can be found in Figure 1 and the resulting list of variables can be found in Figure 2. Note that several of the variables identified in the concept map were not electronically available.]

### Select Measurement Tools

Often times, candidate features can be measured in a variety of ways. Recall the example given above for temperature: it can be measured by digital devices, old fashioned mercury thermometers, or temperature sensitive chemical strips and it can be obtained from the skin, various mucous membranes, the tympanic membrane, the temporal artery, or even from the bloodstream. It can also be recorded continuously or intermittently. Defining how candidate features are to be measured helps one understand potential strengths and weaknesses of the models being built.

[Several candidate features for cardiac arrest prediction were measured by multiple means. Heart rate can be measured by ECG signals or by pulse oximetry. Blood pressures can be measured continuously by arterial lines or intermittently by blood pressure cuff. Temperature can be measured by all the methods described above. As a general rule of thumb, when presented with multiple possibilities, we selected the measurement modality that had the highest reliability. For heart rate, we selected ECG signals. Even by allowing for multiple means of measurement, temperature availability as an electronic source was present in < 10% of the population, so we had to exclude it from the list of candidate features. Blood pressure determinations posed a particular problem, though: there is a difference in data resolution between continuous arterial line readings and intermittent noninvasive (blood pressure cuff) checks. Clinically, when these two measurements disagree, neither is uniformly more accurate than the other. Also, arterial line pressures are not ubiquitous in the population. From a pure "desired properties" standpoint, we felt the noninvasive measurements were more ideal since they are obtained on everyone and since they don't require a procedure to obtain. However, since blood pressure is so fundamental to the concept of shock, and since arterial line tracings provide a more detailed representation of blood pressure, we felt it appropriate to include both modalities in the model.]

### Standardize Candidate Feature Formatting

**Figure 3:**
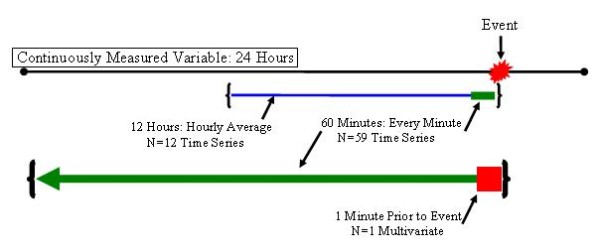
**Data management in the prearrest timeframe**. Multivariable variables were always assigned as the most recent measurement taken before the reference point: the event in this illustration. For continuously measured variables, a multivariable representative was assigned according to the multivariable parameters. The remaining elements were considered time series elements: 59 minute by minute measurements in the one hour preceding the event, and hour by hour measurements in the 12 hours preceding the event.

Time series data are different from multivariable data in one fundamental way: they are repeated measures rather than a single measurement. The timing of their acquisition may be irregular or of an undesired periodicity. In order to use them as features in a model, one must devise a strategy for transforming their native properties into properties appropriate for modeling. This requires several steps, and results in explicit formatting specifications for each of the candidate features. The steps to this process are shown graphically in Figure 3.

#### Determine Class of Representation

The first step of this pricess is to determine whether the feature should be represented in a multivariable format (single value) or in a time series format (multiple values). To make this decision, one must evaluate the tradeoff between potentially useful trend information in the time series format and the complexity it adds to the modeling process. Time series data can be collected in fixed intervals (such as vital signs in physiologic monitors) or they can be collected in nonstandard intervals (such as laboratory measurements). Fixed intervals are somewhat easier to manage, since the predictable timing between measurements allows for consistent representation between subjects. Nonstandard intervals pose the problem of many measurements being taken in a given time period for some subjects with single or no measurements being taken for other subjects. Nonstandard intervals can still be represented in a time series format, but additional specifications need to be determined for how to standardize their representation. Another strategy is to encode nonstandard interval features in a multivariable format, using single values (first, last, mean during some timeframe...) to represent them in the modeling process.

[We represented physiologic monitor data in a time series format and laboratory and demographic data in a multivariable format. Both noninvasive blood pressure measurements and laboratory measurements are characterized by nonstandard intervals of measurement. We treated the noninvasive blood pressure measurements as time series data since their frequency of measurement far exceeded the frequency of laboratory measurements. Many patients in the arrest group had multiple laboratory measurements taken in the hours preceding their arrest, and we were concerned that representing these as time series elements could bias model performance, since the number of unique measurements taken could itself serve as a feature distinguishing arrest from control. Although this could be viewed as a legitimate feature, we felt that the risks imposed by the operator controlled nature of this variable and the potential bias that it would introduce in isolating "time series effects" outweighed the benefits of using it as a feature unto itself.]

#### Identify Reference Point

The second step is to identify a reference point for the time series features so that their relationship to the target of interest is standardized. Typically this will be a particular event (such as cardiac arrest) and measurements can be referenced by the number of minutes that they preceded the event. This strategy would typically be employed in situations where the candidate features lead to the event, which is also the target. If the reference point is an event that may lead to changes in the features, and the target is something else, then measurements can be referenced by the number of minutes that they follow the event.

[We selected the cardiac arrest event as the reference point in time and represented candidate features by the number of minutes that they preceded the arrest.]

#### Specify Windowing Parameters

The third step is to constrain the time series features to a specific window of time and to specify the resolution of measurement within the window. At this step, higher resolution (more frequent) measurements are generally preferable to low resolution measurements, since subsequent steps will use multiple data points in calculations, and higher resolution provides for a more accurate representation of the underlying data than a lower resolution. However, provided there are enough measurements specified in the chosen window to accurately represent any trends of interest preceding (or following) the reference point, the resolution can be reduced from the native resolution of the measurement tool. The resolution does not need to be uniform for the entire window. If trends of interest occur close to the reference point, then measurements taken closer to the reference point can be represented in a higher resolution and those that are farther from the reference point can be represented in a lower resolution.

[Based on our understanding of the physiologic changes that precede an arrest, we chose to include measurements that were taken up to 12 hours prior to the arrest. However, changes in vital signs in the hour before the arrest tend to be more rapid and pronounced than changes that occur greater than an hour before the arrest. In particular, the most dramatic changes occur in the 10-15 minute window before an arrest. For this reason, we chose a resolution of every one minute for vital signs taken in the one hour window preceding the arrest, and we chose a resolution of every one hour for vital signs taken in the 12 hour window preceding the arrest.]

#### Transforming Native Properties to Desired Properties

The final step required to standardize the formatting of candidate features is to transform the native measurement resolution to the resolution specified by the windowing parameters. When the number of native measurements in a given time period preceding or following the reference point exceeds the desired number of measurements specified, a reduction strategy is needed. Several options include selecting the mean, median or mode, maximum, or minimum of the native measurements. When the number of native measurements is less than the desired number of measurements specified, an imputation strategy is needed. Several options include imputing a normal value, a mean, median, or mode value from the data, or carrying forward previous data points.

[We selected the latest measurement taken prior the arrest to represent our multivariable features. The time series features were already represented by every one minute measurements, so no additional transformations were necessary for the one hour window preceding the arrest. Noninvasive blood pressure measurements were an exception. Measurements of noninvasive blood pressures ranged from every one minute to every 60 minutes. Since the corresponding counterparts (arterial line measurements) were continuously measured, we imputed missing values by a simple carry forward strategy and imputed a predefined normal value when no prior measurements were available to carry forward. For the 12 hour window preceding the arrest, the 60 native measurements (every minute) were averaged to obtain the single value specified by the windowing parameter.]

The specific strategy chosen to impute missing data is an important detail. Each strategy involves the creation of new data based on heuristic rather than measurement. As such, each strategy has inherent assumptions, and inherent strengths and limitations. Engels and Diehr present an excellent overview of common practices [85]. In the case of predictive modeling that will ultimately be used in a real-time environment, a strategy that is not dependent on future events is necessary. When imputation must extrapolate beyond known data points, constant measures such as carrying forward last known measurements or using central-tendency measures from previous measurements tend to be more conservative approaches characterized by greater stability over longer periods of time, whereas variable measures (such as from regression analysis) may provide more accurate representations over the short term but the short term benefits are replaced by increased risk the longer these methods are used to impute data [86].

[Our choice of carrying forward most recently measured values was chosen primarily because it is a conservative approach commonly employed in the clinical domain [87]. It has mid-range deviation and bias effects as compared to other available strategies. Our primary goal was to demonstrate the relative information gain afforded by including time series data in the predictive modeling task, so it was necessary to treat both control and arrest groups equally with respect to imputation strategy. We acknowledge that any imputation strategy will differentially affect the two groups if the data is missing in a nonrandom fashion. This area deserves additional study, but is beyond the scope of the current study.]

### Compute Latent Variables

**Figure 4:**
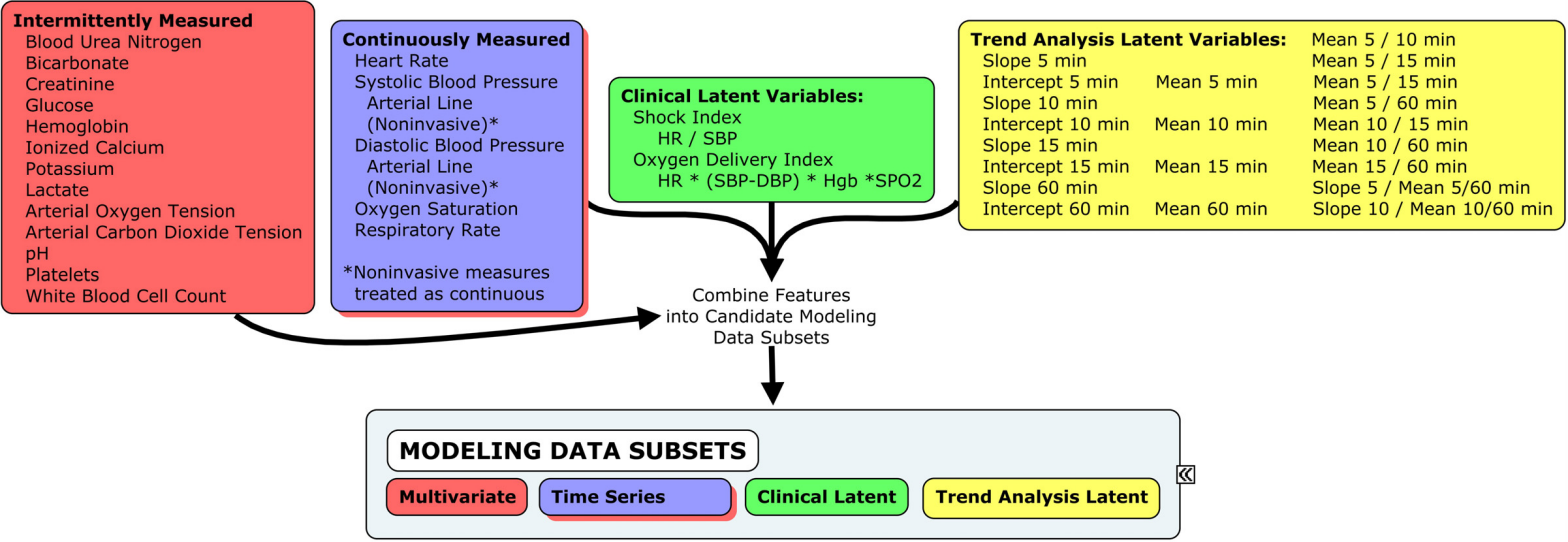
**Data classes**. Latent variables were derived from the raw physiologic Multivariable and Time Series data sets. Clinical Latent Variables were based on calculations used in clinical medicine in assessing for shock. Trend Analysis Latent Variables were based on slopes, intercepts, means, and the ratios of these features for 5, 10, 15, and 60 minute windows that preceded the arrest event.

Although simple inclusion of the raw time series features may be sufficient to proceed to building a model for cardiac arrest, there are explicit computations that may further improve the ability of the models to discriminate one class from another. At least two such types of computations exist: trend features in the time series data, and clinically relevant latent variables that represent concepts not directly encoded in the raw data. The steps to these processes are shown graphically in Figure 4.

#### Clinically Relevant Variables

When two or more of the features can be mathematically combined to express another feature that has an association with the chosen target, explicitly performing the calculation and encoding the new feature as a latent variable may help discriminate between two target classes. Although many of the advanced modeling algorithms may inherently be able to properly classify without the explicit calculation of the clinically relevant latent variable, it may serve as a way to minimize the size of the modeling data set by allowing for elimination of the core features used in their calculation.

[For the cardiac arrest case, two candidate features, the shock index [88] and the oxygen delivery index [89], can be determined from the variables we selected for our analysis. Given that the calculated measures often convey a better representation of shock than any single variable in isolation, we thought it prudent to explicitly include these two latent variables in the modeling process. Shock index is calculated by dividing the heart rate by the systolic blood pressure. Oxygen delivery is estimated by multiplying heart rate, pulse pressure (the difference between systolic and diastolic blood pressures), oxygen saturation, and hemoglobin. Since each of these values is dependent on continuously measured parameters, we treated them as time series data. Of note, at least one other clinically relevant feature would have theoretical utility in a cardiac arrest model model: oxygen extraction index - the difference between arterial and venous oxygen saturations. If blood supply to a tissue bed diminishes, oxygen delivery diminishes, and the amount of oxygen extracted from the arterial blood increases, thereby decreasing the amount of oxygen in the venous blood. Although there is theoretical utility to this measure, obtaining the arterial-venous oxygen difference requires a central line and unless the central line has a venous oximeter, the measurement requires two simultaneous blood gas measurements. We were unable to include this variable in the model since the data to perform this calculation was rarely present in the raw data set.]

#### Trend Features

For each feature, its time series data is represented graphically as the value of the measurement plotted against the resolution of time specified in the windowing parameter. Depending on the nature of the data and the specific trends that one would expect to help distinguish one class from another, candidate trend features should be explicitly encoded as numerical representations by performing the computations necessary to characterize the features of interest. This can include any number of representations, but the slopes and intercepts, and the mean values for various intervals of time relative to the reference point are standard methods of characterizing "trends" and overall status for any given interval. Unless the trends of interest are uniform and precise, a strategy of calculating slopes, intercepts, and means for multiple intervals may provide a better chance to discriminate between classes than a single set of calculations. Additionally, beyond the single determination of slope, intercept, or mean for any given interval, expressing combinations of features, such as the ratio of the mean of one interval compared to the mean from another interval, may provide an even better discrimination.

[Since the trends leading to a cardiac arrest are not well characterized and vary from case to case, we derived multiple permutations of slopes, intercepts, and means for several prearrest intervals. We chose a 5 minute prearrest interval since the majority of arrests demonstrate a more pronounced deterioration in vital signs during this interval. We also chose 10, 15, and 60 minute prearrest interval in order to provide a diverse representation of trends occurring during multiple prearrest intervals. We explicitly represented ratios between mean measurements for each interval to each of the intervals that preceded it: 5/10, 5/15, 5/60, 10/15, 10/60, and 15/60. Finally, considering that steeply negative slopes combined with baseline low means may provide the best measure of discrimination, we divided the slope by the mean for each prearrest interval in an effort to maximize the contrast of such a combination. The specific elements are presented in Table 1.]

#### Seasonality Features

Seasonality features of time series data also hold important information that is not characterized by multivariable data or by trend features of time series data. If such features are of interest, then mathematical representations of their properties are required if they are to be used as representative features. These may come in the form of autoregressive integrated moving average models (ARIMA) or any number of other time series analyses. As in the case of trend features, if the seasonality features are not uniform and precise, multiple analyses may be necessary in order to properly characterize the seasonality features and encode them as latent variables.

[In the case of predicting cardiac arrest, we are only interested in identifying the initial event, not on predicting future arrests based on previous arrests. We therefore did include any features related to seasonality in this study.]

### Partition Data Sets

Once the data have been normalized, they are ready for modeling. Although the entire data set could be used to train a model, the ability of that model to perform in unseen data (its external validity) would be undetermined. Models should not be put into use until their external validity has been determined [90]. There are two ways to establish external validity. The gold standard is to employ the model in a prospective fashion and measure its prediction accuracy over time, noting any degradation that may occur as a result of changing practices or disease prevalence. However, this is a laborious process and typically is not undertaken unless some measure of how well it is likely to perform already exists. Therefore, in order to estimate the external validity, a random portion of samples usually is withheld so that they are never accessed during the training process [91,92]. Determining how many samples to withhold involves a tradeoff: withholding too many samples may degrade the model's performance, whereas withholding too few samples may inaccurately estimate the model's external validity. Depending on how many examples are in the data, between 20 and 50 percent of the samples typically are withheld for validation as the final step in assessing model performance [93,94].

[We randomly withheld 33% of our data as a validation set. Because we had only 103 cases on which to train, and more than a thousand candidate features, we decided that the training set needed to have as many cases as possible. We did not go as low as 20% because we were concerned that numbers of cases would be insufficient to provide a sufficient resolution of accuracy measurement during model testing: one case in 20 equates to a 5% difference, whereas one case in 33 equates to about 3%.]

### Create Modeling Data Subsets

The final step in preparing to generate predictive models from time series data is to determine what actually belongs in the model. The effort that was put into selecting candidate features, specifying their properties and format, and deriving additional clinically relevant and trend features has provided a comprehensive set of candidate features that are suitable for modeling, but all may not be necessary or appropriate for modeling. From this comprehensive set of features, any number of data subsets can be instantiated to answer the question of "Did that step help improve the model?" In order to answer the question, two or more subsets of the comprehensive data set need to be evaluated through a formal modeling process that involves training and tuning on one data set, and measuring their performance in another. Relative performance from one reference set to the other serves as a measure of the utility of the candidate features that differ between them.

**Figure 5:**
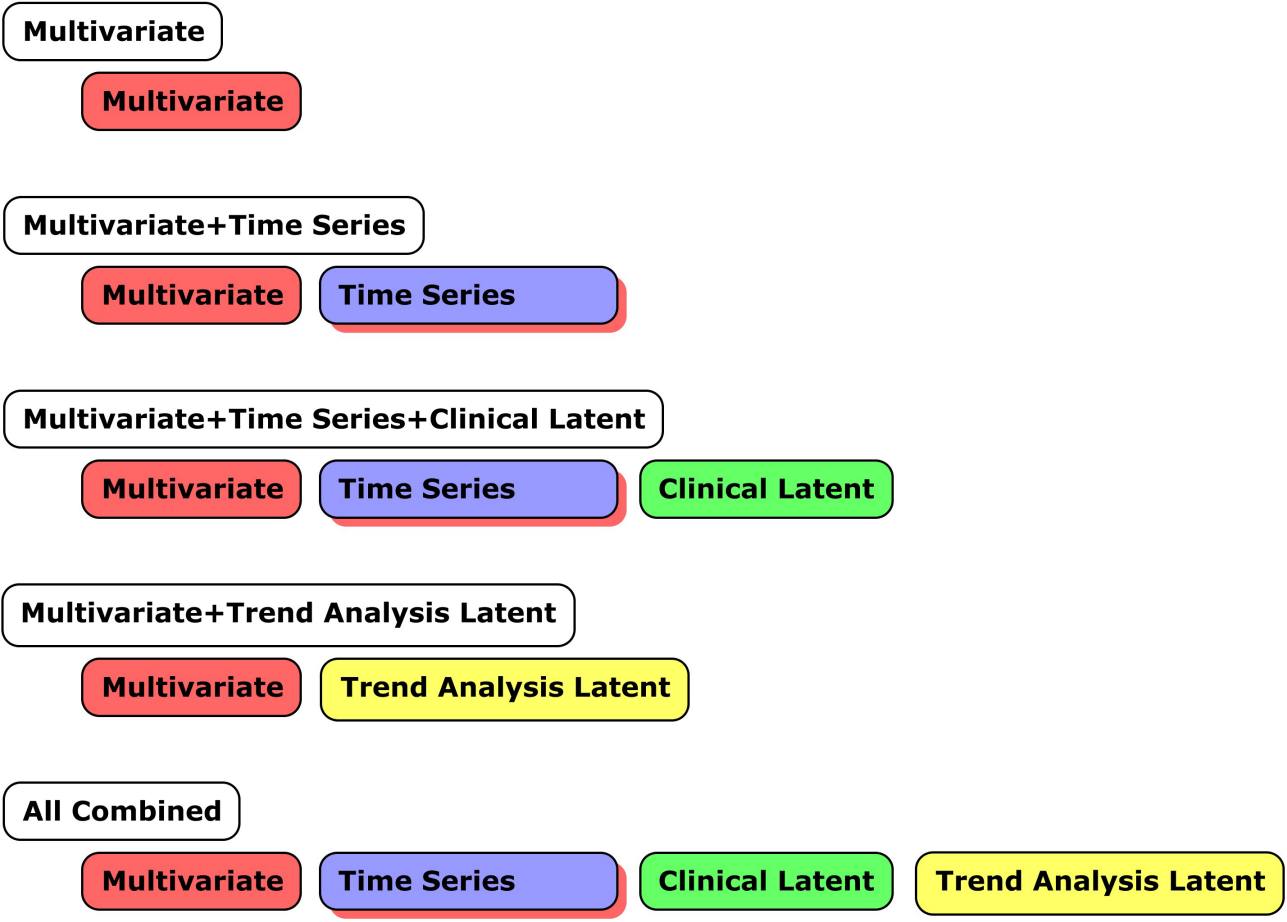
**Data subsets obtained by combining data classes**. Five candidate modeling subsets of data were created to determine the impact of time series and trend analysis latent features (separately) to baseline multivariable model accuracy. Clinical latent variables were compared to multivariable + time series features to determine their relative impact to model accuracy. Finally, all candidate features were combined to determine the net impact of time series + clinical latent + time series latent features on model accuracy.

[Our first task was to construct a data set to serve as a baseline for how well multivariable data alone discriminated arrest cases from control cases. For this baseline data set, we included measurements and physiologically relevant computations that were closest in time to the arrest event. None of the time series features were included in this data set. The second task was to add the raw time series data elements to the baseline data set. The purpose of this model was to determine whether or not the addition of time series elements, without their associated trend calculations, changed the model accuracy. The third task was to add the clinically relevant latent variables with the time series elements to determine whether or not the latent variables provided any additional value to classifying arrest versus control. The fourth task was to add the time series trend features to the baseline data set (without the raw time series elements) to determine whether or not the trend features changed the model performance. The final task was to construct a data set containing all available elements. The purpose of this model was to determine if the combination of time series data elements and their abstracted trend features changed model accuracy beyond any changes that may have occurred by either in isolation. The candidate modeling data subsets are shown in Figure 5.]

### Reduce Candidate Features

Many modeling algorithms are able to discriminate even the subtlest differences between case and control classes of data. When the number of examples is small and the number of variables that the model can use is large, modeling algorithms are prone to memorize the classes [95]. If this occurs, the preliminary results of the model accuracy will be superb, but the model will fail to predict classes accurately in the validation data set. In order to prevent this problem, feature reduction is frequently employed prior to training to reduce the number of candidate variables to a number less than the algorithms need to achieve memorization. As a field of study, feature reduction is broad and has literally hundreds of described techniques that span numerous underlying strategies [96]. At a very basic level, though, feature reduction can be performed by starting with no features and sequentially adding them, or it can be performed by starting with all features and sequentially removing them. Additionally, it can be performed in concert with a modeling algorithm, or independently.

[We selected two strategies that had proven performance in microarray analysis: recursive feature elimination (RFE), which removes features independent of the modeling algorithm, and support vector machine weighting (SVMW), which adds features based on weights assigned during SVM model training. Given that both of these techniques are sensitive to interdependencies among variables for a given outcome (i.e., they will retain two or more variables that work together to make a prediction, even if neither in isolation is highly correlated with the outcome), and that the modeling tool we were using (MATLAB Spider) supported these techniques, we chose them over the simpler, correlation-based techniques. In order to estimate the degree of over-fitting, if any, we also trained candidate modeling data sets without employing feature reduction and compared their performance to those that were trained with feature reduction.]

### Train Models

Once all of the previously described steps have been performed, various modeling algorithms can be used to train models. Before doing so, though, one other point regarding data set management is necessary. So far we have identified two data sets: one for training and another for validation (external validity). Nomenclature in the literature often identifies these as training, testing, and validation data sets [97]. In our methods, we are treating the training and testing data sets a single data set because the modeling tool we have chosen to use can automatically parse and reparse this data set multiple times as necessary to create its own internal representations of training and testing data sets. Many modern modeling tools have this ability to manage training and testing sets automatically from a single data set through use of "cross-validation" routines. These routines essentially split the data set into internal training and testing sets, generate a model and test it internally, and then repeat the process a number of times (known as N-fold cross validation) [97,98]. In repeating this process multiple times using slightly different data sets for training and testing, the models converge on an optimum set of parameters that work the best across multiple splits. This process also generates a measure of internal validity that characterizes how consistent the models are between splits. If models have chaotic internal performance during the training phase, it is somewhat risky to rely on their performance in unseen data as a truly reliable measure of external validity.

As was the case for feature selection, an abundant number of strategies and algorithms can be employed to generate predictive models [99]. Again, at a very basic level, models can be divided into two groups: those that operate with explicit variables and rules and those that operate in a black-box fashion, where the specific mechanism for determining the model output is hidden. To date, there is no single best way to create a predictive model, so several strategies commonly are employed in a trial-and-error fashion in order to identify the optimum strategy for the data at hand. Historically, tools used in clinical medicine have relied on regression-based methods, which work via explicit variables and rules. Decision trees are a class of predictive models that share the same features of explicitness but are based on the more sophisticated algorithms that characterize modern modeling strategies. Although models that use explicit variables and rules provide a degree of understanding to the user as to how a particular prediction is determined, they often do not achieve the same level of raw performance as models that operate in a black-box fashion. Two types of black-box style modeling strategies that have proven to be robust classifiers in multiple domains are neural networks and support vector machines [100].

[Not knowing what the optimum modeling strategy was, we used a combination of candidate modeling algorithms for each of the candidate data sets. For explicit rule based classifiers, we built linear regression and decision tree models. For black-box based classifiers, we built neural network and support vector machine models. Each of the modeling strategies had numerous parameters that could be specified as additional tuning parameters. For the purpose of this study, we accepted default parameters because our goal was to compare various combinations of features and core modeling strategies. Further improvements in performance may have been obtained with an in-depth exploration of parameter effects, but this was considered to be beyond the scope of this study.]

### Validate Models

For any given model that is produced, its performance must be measured in order to demonstrate its utility. Although we alluded above to the distinction between internal and external validity measurements, we did not discuss the specific measures used to assess model validity. As was the case with many of the previous steps, this is an active area of research, and multiple methods of model assessment have been described [95,101].

[For each model generated, we measured classification accuracy, sensitivity, specificity, and the area under the receiver operating characteristic curve (AUROC). These measures of accuracy are well-established and accepted across multiple disciplines, and we considered them to be sufficient to demonstrate differences among the various models we generated. Of note, the ratio of cases to controls in our data set did not reflect the true prevalence of cardiac arrest (the ratio had to be balanced in order for the modeling algorithms to perform optimally). Therefore, positive predictive values and negative predictive values, although listed in performance reports, were meaningless. As a result, other measures that rely on these values (such as gain and the F-statistic) were not evaluated.]

## Discussion

Multivariable models serve as the basis for almost all clinical prediction tools that currently exist. Most of these tools operate on a single value per variable paradigm and are incapable of factoring change over time into their results. Even if the tools called for specific calculations of change over time measures, they would most likely not be used since most of these tools require manual data entry. We have presented a method that allows models to incorporate time series data so that change over time can be factored into their results. In doing so, we have identified a number of practical implications.

We believe automation is necessary in order for any time series based model to be successful and sustainable. Multivariable models and tools can be cumbersome to use, even with only a handful of data points to enter. The number of data points in time series approaches prohibits manual use. Identifying which candidate features to include in the modeling process needs to balance the usefulness of the feature in discriminating between model targets with the ease of getting the feature's measurements into the model. Data feeding the model needs to be populated automatically if the model is to be simple enough to be accepted. Manual data entry of large amounts of data is impractical for sustained model use. These factors need to be considered as specific measurement tools are sought for each of the desired features being included in the mode. Also, the candidate features should be fairly ubiquitous in the study population and part of routine patient care. Although this isn't an absolute requirement, having readily available data facilitates model development by providing a rich retrospective body of data on which to build and tune the model. Data that is rarely obtained has a greater chance of biasing model performance, and will serve to limit the population to which the model is applicable. Likewise, data that requires specialized equipment to obtain or is not part of routine patient care increases the complexity of the overall process since it would likely prohibit a prospective protocol for data collection - again potentially biasing model performance since consenting an entire population of patients is unfeasible. Once data can feed into the model automatically, filtering and processing the data into a standardized format is necessary. The initial determination of the format needs to be informed by a conceptual model that relates the candidate features to the targets of interest. Once the format is determined though, extensive preprocessing must occur in order to transform the raw measurements for each variable into its desired format. The methods chosen to accomplish these preprocessing steps should also be compatible with automation.

The addition of time series analysis results as latent candidate features opens up a realm of possibilities - only a few of which we've covered in this study. We've specifically presented possibilities of using calculations based on clinically meaningful concepts and trend analysis. However, if targets of interest occur repeatedly, then analyses of seasonality features can also be included as candidate features. To date, these types of analyses have received attention in isolation, but have not been used as candidate features with a broader set of variables in a comprehensive theoretically based modeling construct as we have proposed here. This is work to be done.

While current models and tools are typically based on regression strategies with multivariable data, the quantity of data involved in modeling with time series elements requires algorithms that are more robust when ratios of cases to candidate features is small. These tools have made some progress into clinical literature, and several clinical tools are based on them, but their use has still been limited to a multivariable data paradigm. Historically, tools that have relied on black box methods (such as neural networks or support vector machines) have encountered significant resistance for clinical use: the mechanisms used to determine their predictions are hidden and are often nonlinear. The models cannot be shared on paper - they must be run as a program. Since one cannot know a priori how changes in a given a variable will impact the model output, it is difficult to "sell" the model as useful, even though its performance may exceed the performance of models based on a regression formula or a decision tree: being on paper in black and white allows the user to see in advance how change in a given variable will impact the prediction. As more studies show these tools to be superior to their regression based counterparts, we anticipate these advanced algorithms will gain broader acceptance, making clinical acceptance of the methods proposed here more likely.

Finally, we have demonstrated application of this method in a case of cardiac arrest prediction in a pediatric intensive care setting. We hope we have presented each of the steps with sufficient detail that they can be generalized to other scenarios where historical data is used to derive information that helps guide actions. Being able to automatically analyze this type of data and integrate it with other features to arrive at a final determination has inherent potential as decision support that can help end users such as bedside caregivers understand the information contained in the historical data.

## Conclusions

Reviewing predictive modeling paradigms from nonclinical domains provided a potential template for incorporating time series data elements into clinical prediction tasks. The techniques used for time course analysis, which utilizes patterns of measured gene expressions over time to distinguish different biological classes, can be adapted to analyze repeated clinical measures in a similar fashion. We have proposed a six step method for creating clinical prediction models using time series data. The steps include: developing a theoretical construct around a prediction task; selecting candidate features and specifying their properties to match the theoretical construct; preparing data for modeling by transforming it from its raw state into a standardized representation; characterizing clinically relevant calculations and time series analysis as latent candidate features; and creating data subsets to measure the effect that each category of candidate feature has on the predictive accuracy of the resulting model. We have also demonstrated the general process for building and validating models.

We have used progressive shock as a common mechanism leading to cardiac arrest in a pediatric intensive care unit as an illustrative example. A successful prediction model for this phenomenon based on automated techniques could be used to monitor patients continuously for the risk of cardiac arrest. Continuous, objective measures are more likely to systematically identify these children than are the intermittent checks that characterize how they are currently identified. Implementation of such a tool could ultimately serve to save the lives of hundreds, potentially thousands, of children every year in the United States alone.

## List of Abbreviations

We use the following abbreviations in the manuscript: ARIMA: autoregressive integrated moving average; ECG: electrocardiogram; ICU: intensive care unit; PICU: pediatric intensive care unit; PRISM: pediatric risk of mortality.

## Competing interests

The authors declare that they have no competing interests.

## Authors' contributions

CK performed work related to conception, review and synthesis of literature, method design, and manuscript preparation, with oversight by academic mentorship committee throughout.

JT provided critical review and advised the refinement for concept and method development. He reviewed and augmented draft manuscripts.

Both authors have read and approved the final manuscript.

## Author's information

CK is an Assistant Professor of Pediatrics in the Pediatric Critical Care Section of Baylor College of Medicine, working at Texas Children's Hospital. The work presented here constitutes the theoretical background for a PhD in Health Informatics at The University of Texas School of Biomedical Informatics.

JT is an Associate Professor in the School of Biomedical Informatics at The University of Texas Health Science Center, Houston.
